# Morphological communication: exploiting coupled dynamics in a complex mechanical structure to achieve locomotion

**DOI:** 10.1098/rsif.2009.0240

**Published:** 2009-09-23

**Authors:** John A. Rieffel, Francisco J. Valero-Cuevas, Hod Lipson

**Affiliations:** Mechanical Engineering Department, Cornell University, Ithaca, NY 14853, USA

**Keywords:** morphological communication, morphological computation, tensegrity, robotics

## Abstract

Traditional engineering approaches strive to avoid, or actively suppress, nonlinear dynamic coupling among components. Biological systems, in contrast, are often rife with these dynamics. Could there be, in some cases, a benefit to high degrees of dynamical coupling? Here we present a distributed robotic control scheme inspired by the biological phenomenon of tensegrity-based mechanotransduction. This emergence of morphology-as-information-conduit or ‘morphological communication’, enabled by time-sensitive spiking neural networks, presents a new paradigm for the decentralized control of large, coupled, modular systems. These results significantly bolster, both in magnitude and in form, the idea of morphological computation in robotic control. Furthermore, they lend further credence to ideas of embodied anatomical computation in biological systems, on scales ranging from cellular structures up to the tendinous networks of the human hand.

## 1. Introduction

Traditional engineering approaches strive to avoid, or actively suppress, nonlinear dynamic coupling among components. Especially near resonant frequencies, these couplings tend to produce undesirable vibrations and oscillations that are difficult to predict and may sometimes be catastrophic. A variety of passive and active damping techniques have been developed to diminish these effects across many fields ranging from robotics to structural engineering.

Biological systems, in contrast, are often rife with complex dynamics. Consider, for instance, the principle of tensegrity, which can be found at many scales of life, ranging from the cellular cytoskeleton (Wang *et al.* 2001) and the structure of proteins (Ingber 1998) to the tendinous network of the human hand (Valero-Cuevas *et al.* 2007). At every scale, these systems contain the type of coupled mechanical and dynamical linkages which are so assiduously avoided in engineering design. Could there be, in some cases, a benefit to this dynamical coupling?

Here we demonstrate how a highly complex mechanical system can learn to exploit its dynamical coupling as an advantage. In particular, inspired by examples of tensegrity-based mechanotransduction in nature (Parker & Ingber 2007; Valero-Cuevas *et al.* 2007), we construct a highly connected and irregularly shaped tensegrity structure which is able to use the coupling imposed by pre-stress stability as an emergent ‘data bus’ for communication between independent strut modules. This coordination is facilitated by spiking neural networks which are capable of tuning time-sensitive responses to match the particular dynamics of the structures.

This novel demonstration of the emergence of ‘morphological communication’ presents a new paradigm for the decentralized control of large, coupled, modular systems. Furthermore, these results lend credence to the idea of embodied anatomical computation in biological systems.

### 1.1. Morphological computation in biology and robotics

The relationship between morphology and control in biology is a richly studied and fascinating topic. Recent research on the tendinous network of the human hand indicates that the system performs ‘anatomical computation’ in the researchers' words by distributing and switching the tension inputs of the tendon network in order to differentially affect torque at the finger tips. It is conjectured that ‘outsourcing’ the computation into the mechanics of the structure allows related neural pathways to devote their resources to higher level tasks (Valero-Cuevas *et al.* 2007). Similar phenomena have been shown in the physiology of wallabies, whose gaits remain remarkably consistent when switching from level to inclined hopping (Biewener *et al.* 2004), guinea fowl, who exhibit remarkable passive dynamic stability when encountering a sudden drop (Daley & Biewener 2006), and cockroaches, in which muscles within the same muscle group behave alternately as motor and brake (Ahn & Full 2002). Pfeifer and co-workers (Iida & Pfeifer 2006; Pfeifer & Bongard 2006; Pfeifer *et al.* 2007) coined the term ‘morphological computation’ to describe this class of effect. Blickhan has similarly used the phrase ‘intelligence by mechanics’ (Blickhan *et al.* 2007).

We are particularly interested in what we call ‘morphological communication’ in which morphology, physical forces and displacement all act as a non-neural conduit of information, rather than being used to transmit mechanical properties such as power or balance, as in the more general case of morphological computation. Examples of morphological communication can be found throughout biology, such as in the mechanotransduction of the ear (Mammano & Nobli 1993), heart tissue (Parker & Ingber 2007) and with the the neuromechanical modelling of salamander gaits (Ijspeert *et al.* 2007). Morphological communication is closely related to biomechanical passive dynamics (Collins *et al.* 2005), but more specific in the sense that it is understood to explicitly transmit information, rather than simply add dynamic stability to a system.

It is important to clarify that, although it is undeniably the principles of mechanics that cause these functional interactions, there is a strong reason to believe that they likely evolved in response to brain–body coevolution. Namely, that there is (in engineering terminology) a functional advantage for the brain–body system to ‘outsource’ features of control and information transfer to a ‘complex’ anatomy. This is different from simply changing the segment lengths in a limb to grant force or velocity-production capabilities, or evolving the geometry and mass of a system to favour specific limit cycles for passive dynamic function—those would be examples of evolutionary anatomical adaptations, but not necessarily of brain–body coevolution for improved control or information transfer.

Biological morphological computation has served as inspiration for robotic control in several recent works. Iida & Pfeifer (2006) explored how the body dynamics of a quadruped robot can be exploited for sensing. Paul *et al.* (2006) showed how the pre-stress stability of tensegrity robots adds to the robustness of evolved gaits. Watanabe & Ishiguro (2008) demonstrated how inducing long distance mechanical coupling in a snake robot improves its ability to learning a crawling motion.

In contrast to this earlier work, the results which we present in this paper arise from a significantly more complex morphology with profoundly more degrees of freedom. In this domain, morphological computation is not just desired, but essential. We illustrate the tight coupling between our evolved controllers and the system's dynamics in two ways. Firstly, we demonstrate how, when the timing of the gait and the dynamics of the structure are subtly changed, locomotion varies both quantitatively and qualitatively—not just in terms of robustness (Paul 2006) or stability (Iida 2006), but through drastically new gaits. Secondly, and uniquely, we clearly illustrate the emergence of dynamical interactions as a means of communication by observing the coupled behaviour of independent neural network controllers within the structure.

## 2. Tensegrity control and locomotion

It is worth examining the qualities of tensegrity structures which make them an intriguing and challenging platform for robotics. The word tensegrity, a concatenation of tensile integrity, was coined by Buckminster Fuller to describe structures popularized by the sculptor Kenneth Snelson in 1948 (Fuller 1975). A tensegrity structure is a self-supporting structure consisting of a set of disjoint rigid elements (struts) whose endpoints are connected by a set of continuous tensile elements (strings), and which maintains its shape due to the self-stressed equilibrium imposed by compression of struts and tension of strings (Wang 1998). Such structures are pre-stress stable, in the sense that in equilibrium each rigid element is under pure compression and each tensile element is under pure tension. The structure therefore has a tendency to return to its stable configuration after being subjected to any moderate temporary perturbation (Connelly & Back 1998). This pre-stress stability imposes a significant degree of dynamical coupling to the system, in the sense that any local perturbation of a rod causes a redistribution of forces throughout the structure, and a corresponding re-alignment of the struts. Although a qualitative measure, the degree of coupling can be understood to scale with the total number of pre-stressed connections in a tensegrity, as well as the total number of struts.

These properties provide high strength-to-weight ratio and resilience, and make tensegrity structures highly prized in engineering and architecture. Tensegrity can be found in a variety of everyday structures, ranging from free-standing camping tents to the geodesic domes of sports stadiums. Tensegrity structures are becoming increasingly appealing as a medium for smart structures and soft robotics (Sultan 1999; Tibert 2002; Motro 2003; Matsuda & Murata 2006), consequently, recent attention has been paid to their control and manipulation.

Unfortunately, these qualities which make tensegrities so attractive carry with them complex nonlinear dynamics, even for relatively small tensegrity structures (Skelton *et al.* 2001), and as a result, active control is needed to dampen the vibrational modes of relatively modest structures. In almost all cases, deformation and control are achieved by changing the rest lengths of the tensile elements, for instance by attaching strings to a reeled servo motor. In this manner, Chan *et al.* (2004) have been able to demonstrate both active vibration damping and open-loop control of simple structures. Efforts such as these, however, seek to minimize and control the complex dynamics of tensegrity structures, and no effective model exists for the control of the complex dynamics of relatively large tensegrity structures.

More recently, Paul *et al.* (2006) demonstrated an ability to produce static and dynamic gaits for 3- and 4-bar tensegrity robots via evolutionary optimization, and implemented these gaits on a physical robot. Related work demonstrated how the overall stability of these structures results in beneficial resilience and redundancy of control mechanisms (Paul *et al.* 2005). Although these gaits did not seek to suppress the dynamical properties of the structures, and the evolved gaits contained dynamical aspects, the complexity of the structures was relatively low, and the solution relied upon a centralized and open-loop controller.

Rather than attempting to scale these control schemes to arbitrarily large and complex structures, our interest lies in harnessing and exploiting these very same dynamics. We are particularly interested in methods of controlling large, and irregular tensegrity structures—those with much higher degrees of dynamical coupling and complexity than the regular towers of Chan *et al.* (2004) and the minimal structures of Paul (2006).

**Figure 1.**
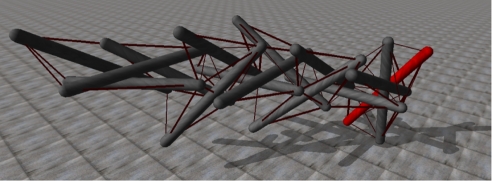
A complex and highly dynamically coupled 15-bar tensegrity structure. High degrees of dynamical coupling is a systemic quality of tensegrity structures.

**Figure 2.**
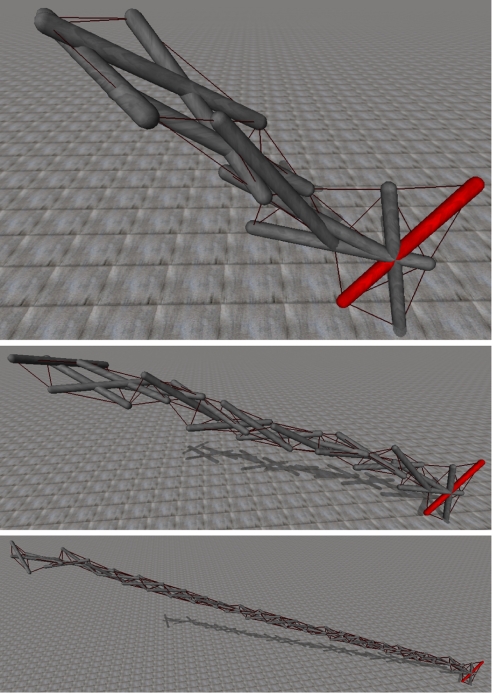
A family of tensegrity towers produced by the same grammar as the tower in figure 1. As the number of iterations of the grammar increases, the tower grows from 10 bars to 20, 30, 40 and 50, repeating the same pattern of twisting bars as it grows.

As a nominal reference structure in which to test our ideas we have chosen the tensegrity in figure 1, which contains 15 rods and 78 strings. This structure is of particular interest because it belongs to a class of tensegrity towers generated by a single generative map L-system which can be scaled, with a degree of patterned similarity, to towers with more than 50 rods, as shown in figure 2 (Rieffel *et al.* 2008).

Such a large and complex structure stymies conventional methods of tensegrity control, and calls for a new paradigm in the control of large, dynamically coupled systems.

### 2.1. Challenges of tensegrity robotics

Constructing robots from tensegrities is a double-edged sword. On the one hand the homogeneity of the rigid elements allows for a high degree of modularity: each rod can contain identical sets of sensors and actuators. On the other hand, any solution which relies upon centralized control of the robot faces a crucial problem: that of communication between modules. As the number of modules increases, the lines of communication (quite literally) increase, bringing both the challenge of coordination and the risk of tangles.

Consider, for instance, the tensegrity shown in figure 1. Even with a single sensor and actuator at each end of each bar, a centralized controller would need to synthesize, and coordinate the actions of, 30 sensors and 30 controllers.

We implement a simpler alternative to the problem of control and locomotion by doing away with the notion of explicit intermodular communication completely, in favour of a more decentralized emergent behaviour (Ishiguro *et al.* 2006; Watanabe & Ishiguro 2008). In our model we consider each rod of the tensegrity to be a simple module with a small controller only capable of sensing and affecting the tension on a single string at each end. We demonstrate how locomotion can emerge by exploiting the dynamic coupling of these otherwise autonomous tensegrity modules. In a sense, the body of the robot becomes an ad hoc network for communication between modules.

## 3. A modular framework for tensegrity robotics

This work stems out of our efforts at creating innovative tensegrity-based robots. Tensegrities are a compelling, if challenging, platform for robotics. One particularly desirable feature is their collapsibility: by relaxing their strings, tensegrity robots can be quickly and easily packed into a small volume for transit, and then quickly re-deployed via string re-tightening.

**Figure 3.**
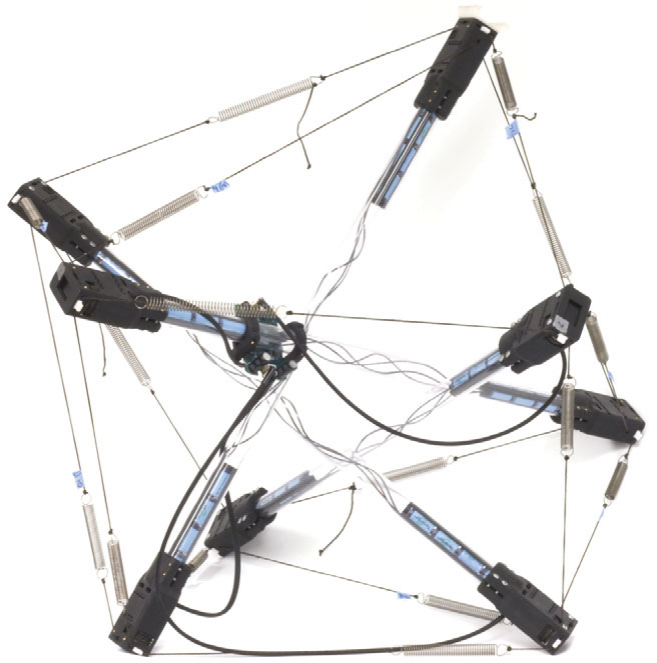
A tensegrity robot consisting of four strut modules and 16 strings. The strut modules consist of servo motors connected by clear plastic tubes which contain batteries and wiring.

Of particular appeal is the highly modular nature of tensegrities, allowing for a significant amount of versatility and reuse: the struts in a 4-bar robot are identical to those in a 16-bar robot. In our design each strut module consists of a rigid tube with a single servo motor mounted at each end. While, in principle, multiple strings could be actuated by multiple servos at each end, we have chosen to keep the design simple by limiting actuation on each end to a single string. Figure 3 shows a photograph of a representative tensegrity robot that contains four strut modules.

### 3.1. Capturing time-sensitive dynamics with spiking neural networks

Since our aim is to embody most of the complexity of a gait within the dynamics of the structure itself, our interest is in a relatively simple controller, such as an artificial neural network (ANN). Unfortunately, conventional ANNs have a critical weakness in this application: they are unable to fine-tune their timing. Consequently, conventional neural networks within each module would require individual hand-tuning in order to find a firing rate at or near the resonant frequency of the structure, and any change in the underlying structure would require re-tuning of the network timing.

In order to add time sensitivity to our structure, we use a variant of ANNs called spiking neural networks (SNNs). SNNs were developed to model more continuous processes: both inputs and outputs are represented as single-value spikes (as opposed to the sigmoid outputs of a conventional ANN; Maass & Bishop 1999). Instead of a sigmoid function, every SNN node contains a simple persistent counter with adjustable offset and limit. At every time step, an SNN node sums its weighted inputs with the current counter value, and if the sum surpasses the limit the node fires a single ‘spike’ to its output; otherwise the contents of the counter are decremented by a fixed decay rate, and persist until the next time step. This ability to fine-tune timing has proven particularly useful in time-sensitive robotics control tasks (Di Paolo 2003).

Each strut module contains a single SNN with two inputs, corresponding to the tension sensed at the single actuated string on each end, two hidden nodes and two outputs. At every simulation time step, each module measures its inputs and feeds them through the SNN. SNN output spikes are then converted into string actuations by measuring the duty cycle of network spikes. Any spike rate above 30 per cent over a 100-step period is considered ‘active’, and the corresponding string is pulled by halving its rest length.

### 3.2. Simulation of tensegrity robots

The representative 15-bar tensegrity shown in figure 1 was reproduced within the Open Dynamics Engine (ODE) Simulation environment, the widely used open-source physics engine which provides high-performance simulations of three-dimensional rigid body dynamics.

Rigid elements were represented as solid capped cylinders of fixed length, uniform mass distribution and a length-to-radius ratio of 24 : 1. Tensile elements were represented as spring forces acting upon the cylinder ends. A given string *s_i_* with length *L*_*i*_, rest length *L*_0_ and spring constant *K* produces a force *F̂_i_*:

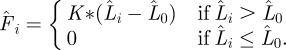



Dynamic and static friction between the struts and the ground is assumed to be infinite and isotropic, corresponding to a rough surface such as carpet. The choice of parameters without our simulation allows us to realistically model the complex dynamics which underly pre-stress tensegrity structures. As we discuss in §5, aware of the ‘reality gap’, we did not fine-tune the dynamics of the model to perfectly match the physical system. The source code for this tensegrity simulator is available for download at the author's website.

### 3.3. Evolving controllers for locomotion

Using this framework, we were able to evolve the weights within the separate SNNs such that the structure as a whole was able to locomote. Each experiment consisted of a population of 150 individuals initialized with random SNN weights evolved over the course of 1000 generations.

With only 30 actuators available (one at the end of each strut module), and a choice of 78 strings to actuate, we chose to evolve both the unique weights of the SNN within each strut module, and also which particular string at each end to actuate. Genotypes of individuals within the population therefore encoded two values. The first contained 180 floating point numbers corresponding to the collective weights of all 15 strut module controllers within the structure. The second consisted of a pairing of actuated strings with strut endpoints. A single point mutation could therefore either change a weight within the SNN or change which string was actuated at a particular endpoint.

Individuals were evaluated within our simulated environment by measuring the travel of the centre of mass over the course of 20 000 simulator time steps. Members of the population were then ranked by their fitness, and the bottom scoring half of the population culled. Seventy-five new individuals were then created as offspring of the remaining population via fitness proportional selection of which 30 per cent offspring were produced with two-parent crossover, and the remainder with single-point single-parent mutation.

**Figure 4.**
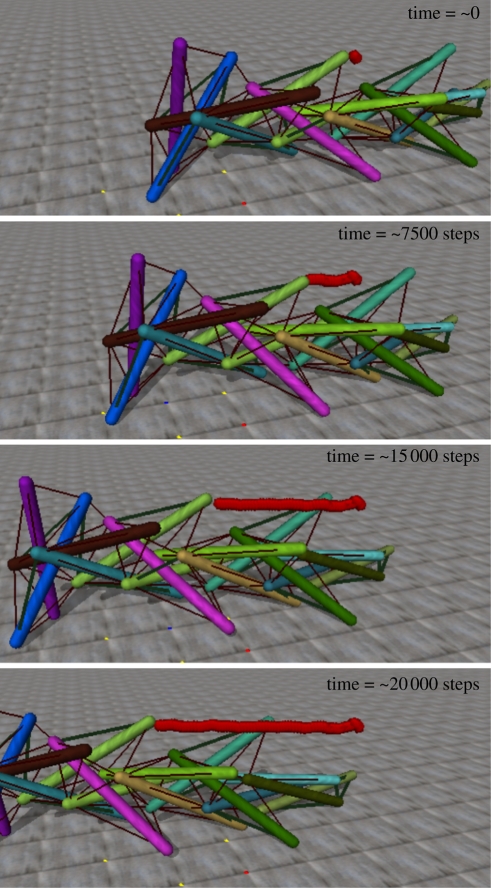
Snapshots of the motion of an evolved gait over 20 000 time steps.

Figure 4 contains snapshots of the movement of one successfully evolved individual over the course of its locomotion. The path of the red sphere above the structure tracks the centre of mass of the structure (vertically displaced for visualization).

### 3.4. A dynamically coupled evolved gait

Since our claim is that the evolved gaits are harnessing the coupled dynamics of the system, we must make efforts to differentiate the results from a quasi-static gait. In a purely quasi-static gait, the movement of the structure and its dynamics are sufficiently decoupled that it can be considered to be stable and consistent over a wide range of speeds. Consequently, neither doubling nor halving the speed of the gait should have a significant effect upon the motion. Consider, for instance, a bicycle wheel which, *modulo* friction, will travel the same distance over five revolutions regardless of the specific angular velocity.

By contrast, in a more dynamic gait, such as a child on a pogo stick, the movement of the system is tightly coupled to its dynamics, and subtle changes in the timing of the gait should have considerable effects on the overall behaviour.

We can therefore qualitatively measure the coupling between evolved gait and system dynamics by observing the behaviour of the structure when the speed of the gait is adjusted by maintaining the system dynamics. Gait ‘macros’ were produced by recording the string actuations caused by the SNNs of an evolved individual over the course of a typical 20 000 time-step run. These gaits were then replayed at speeds ranging from 10 per cent of the normal, evolved rate up to 1000 per cent. Distances covered by the centre of mass over the course of the gait were then observed. The total number of simulation steps was adjusted upwards or downwards accordingly to fit the fixed number of gait cycles.

**Figure 5.**
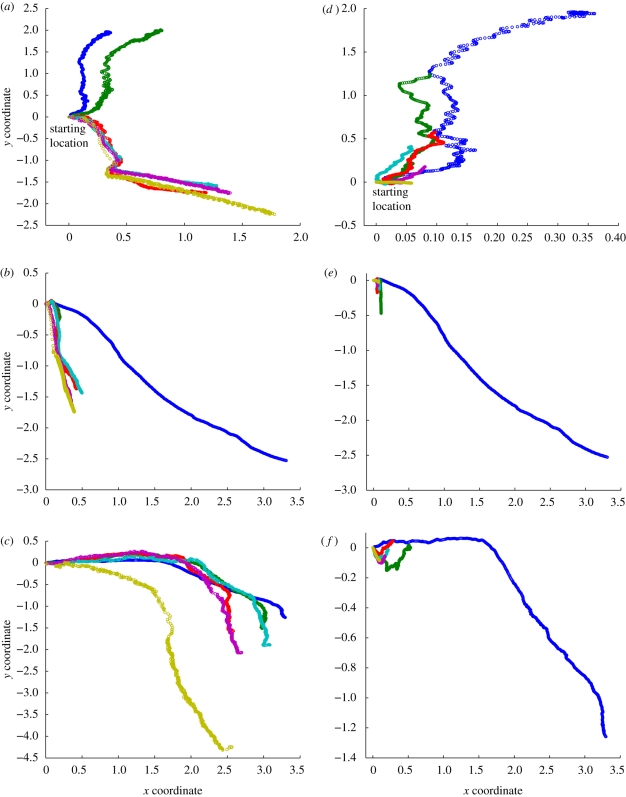
Gait trajectories showing the travel of the centre of mass over the *x*/*y* coordinate frame for faster (*a*–*c*) and slower (*d*–*f*) gait speeds for three evolved gaits (each row). As the speed of the evolved gait changes, both the distance travelled and the path traversed vary significantly. Left- and right-hand figures are not on matched scales. Any increase in distance travelled (slower gaits in left hand figure) is due to the significantly longer amount of simulator time required for the structure to complete a fixed gait cycle. At this time scale, factors such as momentum play a larger role. Blue, full; green, 2 times; red, 3 times; turquoise, 4 times; purple, 5 times; yellow, 20 times.

Figure 5 compares the path of the structure's centre of mass on the horizontal plane over the course of a fixed number of gait cycles for three evolved gaits. Left hand figures contain slower gait speeds and right hand figures contain faster speeds. As is evident, both the distance travelled *and the path traversed* vary significantly under varying speeds.

The degree to which the gaits vary is both significant and surprising, and reveals the deep coupling between the particular evolved gait and the system dynamics. Consider the shift when *Gait 1* varies from one-third to one-fourth of the normal evolved rate: the direction of travel shifts 90°. The remaining gaits also demonstrate significant shifts in trajectory under slower speeds. In general, faster gait speeds by comparison tended to exhibit more pathological changes. In several of the analyses of faster speeds, particularly for *Gait 2* and *Gait 3*, the behaviour of the robot changes so much that the robot collapses onto its side, at which point motion effectively ceases. (It is worth noting that in several cases the robot appears to travel *further* under slower gait speeds than at the normal speed. This is largely due to the fact that the total amount of simulator time in which the gait is observed had to be increased in order to fit a full fixed gait cycle. At this time scale, factors such as the momentum of the structure play a larger role in the overall motion of the robot. If the graphs were normalized for a fixed number of simulation steps rather than for a fixed gait cycle, these slower gaits would of course travel much less far.)

These figures demonstrate a significant behavioural diversity in response to subtle changes in timing, which arises through the interplay between the actuator forces and the intrinsic dynamics of the system. These serve only to show however that the gait is exploiting the dynamics of the system—in the following section we explore the emergence of morphological communication in the evolved controllers.

### 3.5. Morphological communication via dynamic coupling

An equally compelling result is the emergence of de facto communication between independent module networks via the dynamical coupling of the modules. To demonstrate this phenomenon, during locomotion we disable the output of a single module network and observe changes in behaviour in other module networks. Communication between networks can be measured by the degree to which the behaviour of one network affects distal networks.

**Figure 6.**
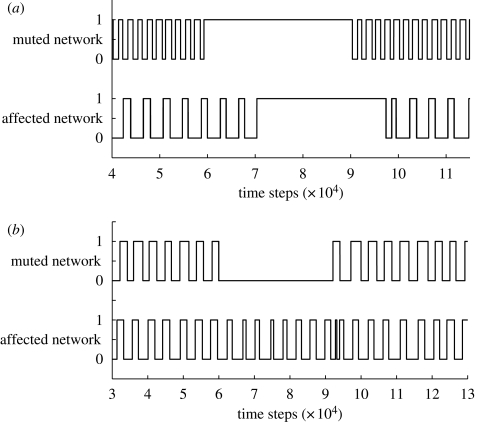
Demonstration of the emergence of communication between individual module networks via dynamical coupling. In (*a*) suppressing the first network causes a distal network to cease activation. Enabling the first network re-enables the second. In (*b*) suppressing the first network causes the second network to increase its firing frequency. After enabling the first network, the distal network resumes the former frequency. The time delay observed between behaviour shifts in the distal networks corresponds to the propagation of dynamics through the system.

Figure 6 demonstrates two such examples of this phenomenon. In the first example, suppressing the output of one module network causes a distal network also to cease activity, and re-enabling the first network also re-enables the second. In the second example, suppressing the output of the first network significantly increases the firing frequency of the second network, and re-enabling the first network results in resumption of the secondary network's original frequency.

Both these are compelling demonstrations of how individual networks can affect the behaviour of distal networks through their shared dynamical coupling. In essence, the morphology is acting as a data bus for communication between module networks.

## 4. Discussion

In approaching the control of tensegrity systems, the conventional engineering approach has been to mitigate and attenuate the complex coupled dynamics and vibrational modes which the structures exhibit. Naturally, as the size and scale of these tensegrity systems increase, and as their regularity decreases, the task of control becomes exceptionally difficult. The tensegrity system we have explored in this work is of a scale and complexity which renders it largely unsuitable for control through conventional means.

Rather than abandon the use of these large, complex, irregular tensegrity structures for novel engineering purposes, for which there is no lack of demand, we have demonstrated how to harness, rather than mitigate, their complex dynamics. By using control schema which are able to discover and exploit the particular dynamics of the structure—in our case SNN—the structure at large becomes, at once, both data bus (distributing information between modules via their coupled dynamics) and powerful actuator (taking advantage of inherent oscillations in order to achieve locomotion). In other words, the morphology of the robot is performing both *computation* and *communication*.

### 4.1. Morphological communication as a paradigm for robotic control

These results present a new method of scalably controlling increasingly complex dynamical mechanical systems such as tensegrity-based robots. We have shown that morphological communication can emerge when a suitable dynamically complex system is married with a suitable responsive control scheme, one capable of tuning itself to the system's dynamics. In the case of our tensegrity work, the complex dynamics are provided by the pre-stress stability which is fundamental to tensegrities, and the time-sensitive control is provided by a collection of independent SNNs.

Several incredibly compelling new questions stem from this work as well. To begin with, more concrete and quantitative means of measuring and analysing morphological communication must be developed. Armed with these, we should be able to make a careful study of how morphological communication scales with the complexity of the system being controlled. Are, for instance, 40-bar tensegrity structures any more capable of this phenomenon, or are 10-bar systems any less capable? Beyond tensegrities, what other complex mechanical systems might lend themselves to morphological communication?

We must also acknowledge that this harnessing of complexity comes at a cost: as observed in figure 5, the systems controlled by our evolved spiking networks are operating at the very edge of control, and are finely tuned to the specific dynamics of their environment. In many cases the evolved controllers seem to have discovered how to walk a ‘tight rope’ of sorts, in that subtle variations in timing can have drastic (and deleterious) effects upon locomotion. Although, on the one hand this would suggest that the evolved gaits do not lend themselves to applications that require a high degree of robustness or stability, it is a compelling thought that somewhat more complex controllers could be evolved which are able to explicitly exploit this behavioural diversity for the purposes of control, for instance by making the robot turn simply by changing the gait frequency.

One final challenge in leveraging these results lies in the ‘reality gap’ between simulated and physical systems. Dynamical effects such as those our solutions exploit are notoriously difficult to model with high fidelity. There are two possible and promising approaches to resolving this challenge in order to create physically embodied robots capable of similar feats of mechanism-as-mind. The first lies in dispensing with the simulator entirely and evolving dynamic gaits *in situ* using embodied evolutionary techniques—such approaches can be slow, but have shown considerable promise (Hornby *et al.* 2000; Zykov *et al.* 2004). The second possibility lies in continuous self-modelling approaches which seek to coevolve robotic gaits alongside an emerging self-model (Bongard *et al.* 2006).

### 4.2. Biomechanics and morphological computation

As well as providing a compelling new paradigm for robotic locomotion, these ideas lend credence to the existence of ‘mechanism as mind’ in the biological realm. This should be of particular relevance to the biomechanics of systems which contain similar mechanical complexities. For instance, given the preponderance of tensegrity-like mechanisms at all scales of biology, the results we have provided offer a compelling new feature of these systems—namely the ability to transmit and receive information via pre-stress stability in order to coordinate action across distal points in the structure.

More broadly, consider the biomechanics of soft-bodied invertebrates such as the *Manduca sexta* caterpillar. Although a well-studied model species, the particular mechanics of *Manduca* locomotion and control remain poorly understood. The caterpillar achieves remarkable control and flexibility despite the fact that each of its segments contains relatively few motoneurons (one, or maximally two per muscle, with approximately 70 muscles per segment), and no inhibitory motor units (Taylor & Truman 1974; Levine & Truman 1985). Studies of the properties of the organism's muscles indicate a high degree of nonlinearity, pseudo-elasticity and strain-rate dependency (Dorfmann *et al.* 2007; Woods *et al.* 2008). It is conjectured that, much like the tendonous networks in the human hand, the complex and coupled dynamics caused by the interaction of hydrostatics, an elastic body wall and nonlinear muscular behaviour are all harnessed and exploited by the organism (via morphological computation) to achieve locomotion in spite of their sparse neural architecture (Trimmer 2008).

## 5. Conclusion

These results demonstrate how the coupled dynamical properties of a complex mechanical system can be exploited for benefit rather than ‘engineered away’. Simultaneously, they lend insight into why biological systems often contain the kind of complex coupled dynamics that are so often assiduously avoided in engineering. It has been conjectured that biological systems which appear under-actuated or under-controlled—such as the tendinous network of the human hand and the body of the *Manduca sexta*—are able to achieve complex behaviour through ‘mechanism as mind’, that is, through the outsourcing of complex control tasks away from the neural directly into the structural mechanics. Here we have demonstrated how such morphological computation can occur in complex mechanical systems, and lend credence to similar phenomena in biological systems.
